# Cross-platform- and subgroup-differences in the well-being effects of Twitter, Instagram, and Facebook in the United States

**DOI:** 10.1038/s41598-022-07219-y

**Published:** 2022-02-28

**Authors:** Kokil Jaidka

**Affiliations:** Department of Communications and New Media, National University of Singapore, Singapore, Singapore

**Keywords:** Human behaviour, Information technology

## Abstract

Spatial aggregates of survey and web search data make it possible to identify the heterogeneous well-being effects of social media platforms. This study reports evidence from different sources of longitudinal data that suggests that the well-being effects of social media differ across platforms and population groups. The well-being effects of frequent social media visits are consistently positive for Facebook but negative for Instagram. Group-level analyses suggest that the positive well-being effects are experienced mainly by white, high-income populations at both the individual and the county level, while the adverse effects of Instagram use are observed on younger and Black populations. The findings are corroborated when geocoded web search data from Google is used and when self-reports from surveys are used in place of region-level aggregates. Greater Instagram use in regions is also linked to higher depression diagnoses across most sociodemographic groups.

Social media platforms are increasingly crucial in studying online communication and self-presentation. As individuals curate their online selves, they aim to exaggerate positive emotions^4,20^ for the benefit of their imagined audiences^15,38^. Thus, a feedback loop of consistently seeking and maintaining social validation ensues, which has implications for the psychological well-being of an individual. Raising these concerns, many scholars have reported the association of social media platform use with subjective well-being and mental health indicators. A variety of methods, contexts, and treatments have been considered. However, conclusive evidence for or against the portended adverse effects of social media remains elusive.

This debate remains in flux for two main reasons. The first reason is the limitations of survey methods in terms of representativeness and scale. Most findings regarding these associations have relied on survey responses by adolescents and students in school and university settings (for a comprehensive literature review, please see Liu et al.^34^, Schemer et al.^49^, Orben and Przybylski^41^, Best, Manktelow, and Taylor^7^). Endogenous variables—such as socioeconomic class, age, race, and gender—associated with social media use and well-being could explain all reported associations. However, there is limited evidence that these findings generalize to adult, multi-ethnic populations with socioeconomic differences. A few exceptions^2,5,23,25^ have explored the role of age^25^, gender^42,57^, and race^23^. They have also highlighted the need to reconceptualize how social media behavior is understood and measured^5^. This study begins with analyzing general social media use with region-level and individual-level data. However, the heterogeneity in the results can often be based only on a region-level analysis, which survey data may not always support. Therefore, the primary findings relate to the platform-specific and inter-group differences in the well-being effects of social media use at the regional level.

The second lacuna in previous work is the limited theoretical explication of social media use. Social media use has often been operationalized as the absolute exposure to social media^41,55,57^. However, recent findings suggest that some platforms appear to be conducive to well-being, while others are not^3,11,34,34,44,58^. Scholars have recommended that analyses should distinguish social media behavior based on types of interactions to provide more insight into the mental health and well-being outcomes.”^11,34,50,59^.

This study confronts the limitations in prior work and addresses the following research questions:

- What are the platform-specific differences in the associations of social media use and well-being?
- What are the inter-group differences of these effects?

Additionally, this study is interested in exploring whether these findings generalize in analyses of other datasets comprising systematic variations in data sampling, the measurement of social media use, and even the mode of data collection. The following additional research questions are posed:

- Do the well-being effects of social media use generalize to analyses with digital trace data?
- Do the inter-group differences in the well-being effects generalize to analyses with individual-level data?
- Do the well-being effects of social media use generalize to other mental health outcomes?

The questions are addressed through the multi-level modeling of regional- and individual-level data from representative surveys by the Simmons National Consumer Survey, Gallup-Sharecare Well-Being Index, and Pew Research, together with Google Trends web traffic data. The primary dataset constitutes region-level consumer trends of social media use collated by the Simmons National Consumer Survey. The primary findings are triangulated with the secondary dataset, comprising geocoded web search data collected from the Google Trends API, and the tertiary dataset, comprising individual-level survey-based findings over 5 years. Together, they enable an understanding of the population-level differences in the well-being effects of social media in the United States. Potential ecological fallacies have been addressed with appropriate controls, cross-lagged predictions, and clustered random effects in all analyses.

## Background

Subjective well-being is a measure of how people evaluate their lives^17^. Although the possible link between social media use and well-being remains a public health concern, the empirical evidence remains inconclusive and suggestive of a marginal or a null effect^14,34,41,42,49^.

The reinforcement hypothesis suggests that online communication can reinforce the quality of existing friendships and enhance well-being^58^. A similar phenomenon has often been reported in the political engagement literature, where the technology may reinforce the offline gaps between those with better and poorer resources^8^. Personality and pre-existing stores of social capital^6^ may moderate the impact of social media platforms on psychological well-being. It can also be anticipated that the quality of online ties^10^ and the tone of social support^53^ predict the relationship of social media use with well-being. A survey of American social media users examined whether social media induced more stress than it relieved^24^. It was found that women reported being more stressed than men, but greater use of Twitter was associated with lesser stress. Women were also more likely to socially share their positive and negative emotions on social media. They speculated that women could take advantage of Twitter as a coping mechanism, where they perceived higher levels of social support, which in turn moderated or reduced their stress.

A competing perspective is the displacement hypothesis, which argues that the time spent on social media displaces time spent with existing friends with interactions of ‘poorer’ quality^13,58^. Online interactions may be poorer if they occur with weaker rather than stronger ties^10,59^, possibly leading to negative implications for self-appraisal and well-being. They are also considered to be poor if they trigger negative affect during or after the interaction^13^. The study by Davila et al.^13^ reported no association between social networking use and depressive symptoms; however, consistent with the findings of Valkenburg et al.^59^, they found that “young people who reported less positive and more negative interactions reported greater depressive symptoms concurrently and over time.” (pp. 10). Poorer quality interactions affect a social media user’s sense of self. For instance, a previous study on the General Social Survey data^25^ reported that the likelihood of nervous breakdown was associated with the number of platforms that respondents used, which was moderated by their age. These findings corroborated those by Primack et al.^45^ on an adolescent population, who reported a linear association between the number of platforms used and depression and anxiety, and the association remained robust after controlling for the total time of social media use. It can be anticipated that using a greater number of platforms may trigger a sense of identity diffusion, which would explain the decrease in well-being^37^. Finally, poorer quality interactions would also include those which encourage upward social comparison. Viewing selfies and other photos on Instagram and Facebook has been related to a loss of self-esteem^61^ and a negative body image^21^. Social media users view posts depicting a heightened positivity bias; therefore, they are also likely to overestimate others’ successes and experience lower affect^60^, envy^45,54,60^, and depression^54^. However, other findings from pre-registered experiments have suggested that upward social comparisons may not always be a bad thing. Meier et al.^39^ suggested that viewing nature and travel posts on Instagram can facilitate benign envy and subsequently enhance well-being.

Because of evidence supporting both hypotheses, it is perhaps necessary to eschew a Manichean perspective about social media effects. Instead, I anticipate that the differential effects of social media use could be interpreted in terms of the differences in their affordances. Each platform foregrounds different affordances or action possibilities to communicate with others or engage with content. Evidence suggests that social media users appreciate these differences and adjust their in-platform behavior accordingly. Platform differences may explain why users are more active on one platform than another^12^. Differences in platform affordances are associated with the cross-platform differences in self-disclosure on Facebook versus Twitter of the same panel of American social media users^27^. Individuals are also reportedly cognizant of the social capital advantages of some platforms vis-a-vis others^44^.

In summary, the way individuals engage with social media is influenced by various technical, social, and communicative factors, which affect both the user and the in-platform behavior of other users. However, most prior work examining the well-being effects of social media use has considered a single, overall measurement of usage or visit frequency. Instead, this study posits that different social media platforms could have different well-being correlates. Therefore, following the paradigm discussed by Bucher, Helmond, et al.^9^, this study focuses on the perceived social and communicative affordances of different social media platforms—specifically Facebook, Twitter, and Instagram—and their importance for well-being.

### Identifying cross-platform differences in well-being effects

Prior meta-analyses^34^ have reported different associations of SNS use with well-being depending upon use type. Individual studies^10,30,60^ have also found different impacts of well-being depending upon type of use. The reader would note that *all social media platforms arguably provide similar uses and gratifications*. However, some platforms appear to be conducive to well-being based on prior findings, while others are not. Why could this be? Considering technological design offers a way to understand the substantial well-being effects and how they differ across platforms and populations—an avenue not explicated in uses and gratifications studies across platforms. Furthermore, understanding social media use from an affordance perspective focuses on the systemic differences in social media platforms that relate to users’ ability to communicate, socialize, or self-present on them. A monolithic perspective on social media platforms ignores the complicity of technological design and the role of platforms in engendering specific uses of their website—for instance, “social networking behaviors that do not fulfill needs for acceptance and belonging” [11, pp. 33] and hence correspond with a decrease in well-being.

Accordingly, this study has separately considered the well-being effects of Instagram, Facebook, and Twitter in region-level analyses of well-being. This choice is premised on the different social, and communicative affordances of each platform, where heightened interpersonal communication and disclosure is encouraged and afforded through Facebook^3,19,27,44,64^ whereas a more isolated and passive experience is afforded on Instagram^34^. Public self-presentation strategies are preferred instead^44,51^. The affordance of unidirectional friendship connections implies that one’s social network may comprise strangers, limiting the possibilities for intimate interpersonal sharing behavior^33,36,62^.

Scholars have raised issues with measures that rely on self-report data to measure exposure^22,46^. Therefore, the primary findings were replicated with a different region-level social media usage measure based on web searches. Previous studies have also demonstrated the use of Google Trends data as a valid approach to measure and predict physical and mental health^28,40^. They have also been used to better understand the patterns of information consumption of Americans^48^. Addressing the limitation of self-report data, Google Trends also offers a proxy to web traffic that can triangulate results based on survey responses^32^.

**Figure 1:**
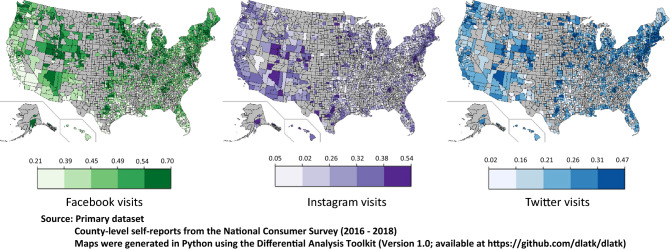
The average county-level relative visit frequencies to Facebook, Twitter, and Instagram across the United States, 2016–2018. Counties with darker shades had relatively higher self-reported visits. Maps were generated in Python using the Differential Analysis Toolkit (Version 1.0; available at https://github.com/dlatk/dlatk).

Figure 1 illustrates the spatial variation in the relative visit frequencies to Facebook, Twitter, and Instagram based on the Simmons self-report data at the county level. Counties with a darker shade had relatively higher visits to the respective platform. Comparing the map for Facebook and Instagram suggests a demographic divide between counties that frequent either platform.

## Results

A sanity check was first performed to compare the well-being effects of general social media use (operationalized as the frequency of social media visits) with the corresponding effects in the primary dataset, comprising region-level survey trends from 2013–2018, against the tertiary dataset comprising individual-level self-reports from 2008–2013. With both datasets, after controlling for sociodemographic and exogenous variations, a 1% increase in social media use predicts an increase in well-being (0.6% rise in well-being in the primary dataset, $\beta = 0.64$, $\hbox {p} <0.01$; 0.08% rise in well-being ($\beta = 0.08$, $\hbox {p} <0.001$). The supplementary materials reported the detailed results, and the year-on-year consistency in the effects is also confirmed.

### Platform-specific social media use and well-being

**Figure 2:**
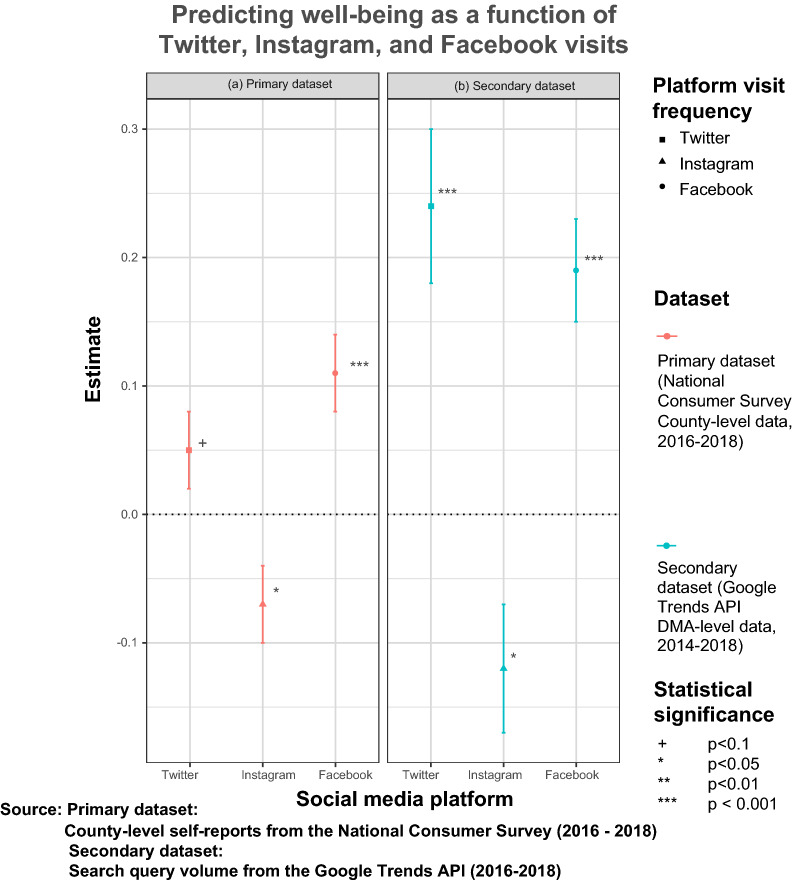
Estimates of the association between platform-specific social media visit frequency and well-being with (**a**) the primary dataset at the county-level, and the (**b**) the tertiary dataset at the DMA-level. All models controlled for sociodemographic variables, and cross-lagged and regional fixed-effects.

The platform-specific effects of social media use (operationalized as the frequency of social media visits to Facebook, Instagram, and Twitter, respectively) for 2016–2018 are reported in Fig. 2a. Before 2016, NCS did not allow respondents to indicate how often they visited each platform. All models included sociodemographic controls and cross-lagged and county fixed-effects. The tabulated results are reported in the supplementary materials.

First, considering the effects based on the primary dataset, a 1% increase in the frequency of Facebook use at the county level predicted 0.11% higher well-being ($\beta =0.11$, $\hbox {p} <0.001$). A 1% increase in the frequency of Instagram use predicted a 0.07% decrease in county-level well-being ($\beta =-0.07$, $\hbox {p} <0.05$). Conversely, the increase in the frequency of Twitter use predicted lower well-being, although this effect is only marginally significant ($\beta =-0.05$, $\hbox {p} <0.1$).

### Triangulating survey data methods with trace data

The platform-specific findings from Fig. 2a are compared against the corresponding estimates based on Google search query volume reported in Fig. 2b for the same period (2016–2018). Once again, all models included sociodemographic controls and cross-lagged and DMA fixed-effects.

The findings bear out the initial results, as a 1% increase in the frequency of Facebook searches (a proxy for use) in a Digital Marketing Area (DMA) predicted a 0.19% rise in well-being ($\beta = 0.19$, $\hbox {p} <0.001$). Likewise, a 1% increase in the frequency of Twitter searches predicted a 0.24% increase in well-being ($\beta = 0.24$, $\hbox {p} <0.001$). Finally, a 1% increase in the frequency of Instagram searches in a DMA predicted a 12% decrease in well-being ($\beta =-0.12$, $\hbox {p} <0.05$). The data window is deliberately constrained according to the availability of the primary dataset, but analyses for a longer period (2014–2018) also bore out similar effects.

### Population differences in well-being effects

Some scholars have evinced concern that adolescent females constitute an especially vulnerable group that bears the brunt of the ill effects of screen time and social media use^56,57^. Although the race differences in socioeconomic status^1^ and stress^25^ have been studied elsewhere, their implications for social media effects have not been explored for group-level differences in social media effects.

**Figure 3:**
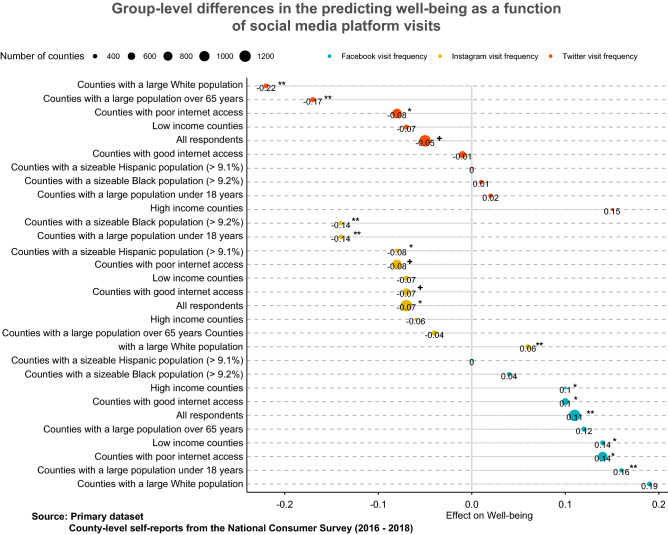
Primary dataset: Subgroup differences in the association of social media use with well-being (2016–2018, NCS data at county-level). All models are cross-lagged and include all other covariates (age, gender, race, income, internet access quality, education) except the one that defines the subgroup.

Following these concerns, the data were reanalyzed at the group level. The findings reported in Fig. 3 identify the group-level differences in the association of social media use with well-being at the county level, based on the primary dataset. The tabulated results are reported in the supplementary materials.

First, consider the effects of Twitter use on well-being across different demographic groups reported at the top of the Figure. The well-being effects are negative for three out of the nine demographic groups, while they were marginally significant with a negative magnitude for all groups. Twitter use predicts negative well-being in counties with poor internet access (counties with broadband not available) ($\beta =-0.08$, $\hbox {p} < 0.05$), counties with a large population over 65 years (counties in the top quantile of a senior citizen population) ($\beta =-0.17$, $\hbox {p} < 0.01$), and counties with a large White population ($\beta =-0.22$, $\hbox {p} < 0.001$).

Next, consider the effects of Instagram use on well-being reported in the middle of the Figure. The trends are steadily negative, except that counties with a large White population (counties in the top quantile of the population of Whites, as per the National Census figures) report a 0.06% increase in well-being with a 1% increase in Instagram use ($\beta =0.06$, $\hbox {p} < 0.01$). On the other hand, Instagram use by all other demographic groups predicts a decrease in well-being ($-0.14<= \beta <= -0.04$).

Finally, the effects of Facebook use on well-being are also homogeneous, as seen in the bottom of the figure, where the increase in Facebook use is typically associated with a statistically significant increase in well-being ($0.04<= \beta <= 0.19$).

A group-level analysis of individual-level data replicated the effect magnitudes, significance, and direction of the relations and is reported in the supplementary materials.

**Figure 4:**
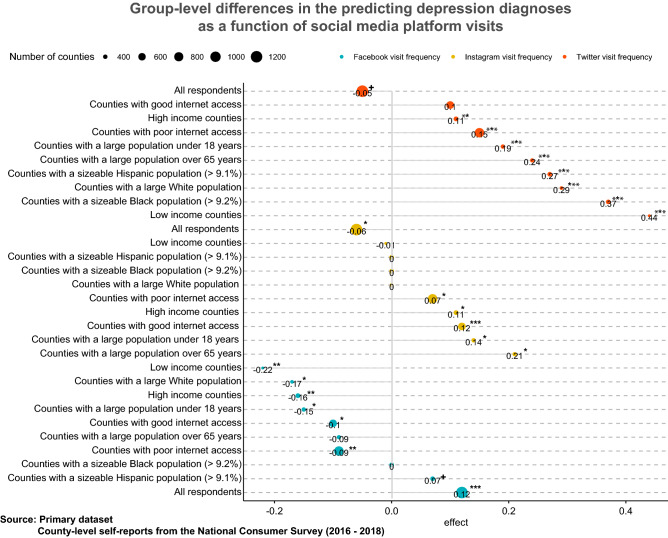
Primary dataset: Subgroup differences in the association of social media use with depression diagnoses (2016–2018, NCS data at county-level). All models are cross-lagged and include all other covariates (age, gender, race, income, internet access quality, education) except the one that defines the subgroup.

### Generalizability to other mental health outcomes

While the findings so far suggest the grave implications of specific social media platforms for well-being, arguments may be raised regarding the long-term dangers of social media use or the tangible mental health implications. Therefore, I replicated the group-level analysis against a measure of the year-on-year county-level prevalence of depression diagnoses as collected by the Gallup-Sharecare Well-Being Index, which is reported in Fig. 4.

As before, higher Twitter use predicts higher depression diagnoses (i.e., worse well-being) across most demographic groups. Higher Instagram use predicts higher depression diagnoses in younger and older counties, but the effects are not specific to ethnic groups. The trends for Facebook are stable as before, as it predicts lowered depression diagnoses across most demographic groups.

## Discussion

Using geocoded digital traces to triangulate small-scale surveys can alleviate concerns regarding the quality and representativeness of self-report data. In addition, similar triangulation approaches can leverage digital traces in any situation where data accuracy may be a concern.

The positive association with well-being does not generalize to all platforms. Instead, the results support the positive effects of Facebook use and the adverse impact of Instagram use on well-being. Note that the effects of usage on well-being are larger than in previous literature because of the aggregation effect of conflating individual-level behaviors to regions.

The differences potentially arise because of the social and communicative differences in how people use Facebook vs. Instagram. Previous studies have also established that users perceive that Facebook encourages intimacy and private self-disclosure^27^, while Instagram emphasizes a positivity bias, emphasis on self-presentation^62^ and communication with weak ties or strangers^36^. Further studies disentangling these effects could incorporate users’ perceptions of these affordances as the dependent variable^5^.

The results from a group-level analysis support the reinforcement hypothesis, with technology widening the divide between those with greater advantages in the offline setting, who can leverage social media for personal benefit. By and large, the findings suggest that white, high-income populations experience the positive effects of social media on well-being at both the individual and the county level. Validation with individual-level data from 2008–2013, when the primary social media platform used in the US was Facebook, bears out the region-level analyses. Facebook is a platform for relatively intimate social exchanges in a network of people known to individuals in offline settings. The results reaffirm scholarly concerns about (counties with) young populations being at greater risk with social media use—specifically, Instagram use. It also heightens the concerns about the impact of social comparison on ethnic minorities. The negative effect of Instagram use on well-being is driven by counties with a sizeable Black population, and an opposite trend is reported for counties with a large White population. The insights are also crucial for social media companies to consider how their design features and algorithms might inadvertently sideline minorities from benefiting from their time on Instagram.

The findings for Twitter users are more nuanced than for Instagram, with an overall negative marginal effect on well-being. The effects swing widely between high-income counties, which experience a net positive effect, and what appear to be rural counties (counties with a large white population; counties with a large population over 65 years; counties with broadband not yet available). Prior surveys have reported how the typical user base of Twitter is urban, professional, and middle-aged and uses Twitter for professional networking^43^; therefore, it would appear that its use by individuals outside this privileged group may predict lower well-being.

When the analysis was replicated with a measure of the year-on-year county-level prevalence of depression diagnoses, greater Instagram use predicts higher depression diagnoses across most demographic groups, while increased Facebook use was predictive of a decrease in the numbers of depression diagnoses at the county level across various demographic subgroups. The demographic results are also differently signed than the aggregate results reported for all respondents, highlighting the need to interpret them at the level of demographic groups and their social environments.

## Conclusion

This study offers spatiotemporal insights into the relationship between social media and internet use, and community health, through a combination of public and restricted data. The findings support the reinforcement hypothesis in understanding how offline divides in well-being are reinforced digital spaces. The research design addresses fundamental questions that are important to the computational social science research community, which actively explores how digital footprints—the fine-grained, time-stamped records of millions of individuals—can be used to understand personal and social identity and communicative behavior. The findings highlight the need to examine how technology design can affect its users. The interface of each platform foreground specific affordances to its users while obscuring others. When Facebook, Twitter, and Instagram users log in, they see a ‘broadcast’ feed with the latest updates on their friends in the front and center of their screen. The Facebook feed is preceded by a ‘nudge’ in the center top, which encourages them to share their thoughts with the message ‘What’s on your mind, X?’: a soft intervention especially designed to promote healthy self-disclosure^19^. It is also overlaid with a popup on the bottom right with a Messenger, encouraging them to engage with their social circle one-on-one with a ‘New message.’

On the other hand, the communicative affordances of Instagram have been hidden away behind an icon on the top right. Opportunities to engage with their network through their posts are limited by default, hiding comments in a feed and requiring extra clicks (or taps on mobile devices) to read one or more exchanges underneath each post. Even when users comment on their friend’s posts or Stories, these are automatically posted and hidden (in the case of Stories) to favor an uninterrupted consumption experience instead of engaged one-on-one conversation. With the affordances to contact friends being hidden away, Instagram makes the user’s experience more isolated and passive—an indicator of harmful social media use predictive of lower well-being^34^, thus supporting the displacement hypothesis. Furthermore, the visual aesthetics of content would encourage self-promotion while making it more difficult to strike up meaningful social connections critical for well-being. The features aside, repeated exposure to self-promotional content on Instagram has been related to upward social comparisons and lower self-appraisal in studies involving adolescents. There is reason to suspect similar negative consequences for an adult population, even if the data only supports inferences at the regional level. Indeed, the data do not allow causal claims. Although the SCNS data analyzed may not have assessed the same participants over time, region-level analyses with cross-lagged models can correct potential auto-aggressive effects. Second, there is potential that omitted variable biases may have crept in where variables representing population density, crime rate, and comorbidities are not included in the analyses. Including education and income statistics has potentially assuaged the omitted variable bias. From a body of work in well-being^16,18^, it is known that socioeconomic differences drive differences in well-being, health, crime, and many other metrics of regions and societies. The ecological fallacy plays a role in inflating region-level estimates, and these concerns have been raised elsewhere^26^. Finally, validation with depression diagnoses highlights how the proportion of the at-risk population is higher in counties with higher social media use but does not imply that using social media contributes to depression diagnoses.

In summary, this study suggests that in the United States, the technological design of social media platforms can affect their users. The availability of granular, temporal, demographic, health, and social media usage data from other countries could facilitate a replication for other countries, where the effects may be amplified in countries with higher social media use, such as India and Brazil, or attenuated where social media penetration is lower, such as Japan.

The findings suggest that the harmful effects of social media may be systemic and should be studied by considering and not obscuring their perceived and actual infrastructural capabilities. Furthermore, the findings offer grounds to believe that the affordances that enable, constrain, and control social and communication norms that evolve on these platforms could also provide more nuance in understanding the relationship of technology to behavior and well-being. The policy potential and implications of digital data for society are worth considering, as they promise applications for civic life and healthcare^35^ without necessarily compromising privacy^47^. In future work, scholars can consider using experimental methods to establish causality and explore other exogenous factors that could disentangle the myriad ways social media use impacts societies.

## Methods

### Data

#### Primary dataset: county consumer data

Average county-level data on social media use between 2013–2018 was collected from the Simmons National Consumer Survey (NCS), available by license. NCS is a cross-sectional survey that generates annual county-level estimates by weighting observations to match the demographic profile of the American public. Until 2015, NCS collected information on general social media visits. For 2016–2018, NCS collected separate usage statistics for the weekly and monthly use of Facebook, Twitter, and Instagram. While the SCNS data is granular, it may suffer from other issues, such as ceiling effects. I address this issue by operationalizing a social media use metric that combines two separate survey items related to the visit frequency for Facebook, Twitter, and Instagram. Before analysis, all social media visits information was rescaled to a value between 0 and 1.

Following prior operationalizations of county-level well-being and depression^26^, over 1.5 million responses to the Gallup–Sharecare Well-Being survey over 2014–2018 were aggregated and averaged to obtain yearly county-level measurements of the Well-Being Index (2014–2018) and depression diagnoses (2014–2018). Only counties with at least thirty responses collected in a single year were considered, which resulted in county-level measurements for 1216 counties. This data was available by license to the university of the first author. In addition, sociodemographic correlates related to the age, gender, and race distribution, percent with a college education, and the average household income for each county are taken from the American Community Survey (ACS). Exogenous variations in social media access are accounted for by using data about broadband rollout (Digital Subscriber Lines or DSLs installed) information from the Federal Communications Commission (FCC). The download speed is operationalized as a binary variable reflecting the presence or absence of DSL lines among at least 80% of the county residents.

#### Secondary dataset: Digital Marketing Area (DMA) web search data

Self-report data has previously been suggested to be an unreliable measure of exposure^22,46^. Therefore, the main findings were validated with a third dataset comprising the yearly search query volume for 2014–2018 for different social media platforms, to match the years for which the primary data was available. Year-on-year geocoded Google Trends data at the Digital Marketing Area (DMA) level about Facebook, Twitter, and Instagram searches were collected for each DMA (n = 205). A greater search volume for social media platforms would indicate a greater number of visits to either platform.

Responses from the Gallup-Sharecare Well-Being Index between 2014–2018 were averaged to DMA-level rather than county-level by mapping FIPS codes into DMA zones based on documentation used elsewhere. In addition, Internet quality was based on information from the FCC regarding the availability of broadband at the county level, mapped to DMA zones.

#### Tertiary dataset: individual-level data from Pew Research

The relationship between well-being and general social media use has been traditionally examined at the individual level. To establish the validity of the findings at the region-level, I corroborated the spatial analysis with findings based on individual-level, general-social media usage and life satisfaction, collected earlier than the primary and the secondary data samples (2008–2013) as part of the Pew Tracking Surveys (n = 16,064). Details about the questions and the measurement variables are provided in the Supplementary Information.

### Analytical framework

For a preliminary understanding of the spatial variation in social media visit frequencies for Twitter, Instagram, and Facebook, choropleth maps were generated using the primary data as input, using Python code available as a part of the Differential Language Analysis Toolkit^52^.

The platform-specific differences in well-being effects in the primary dataset were examined for 2016–2018. The findings were validated with the secondary dataset comprising search query volume data collected from Google Trends at the Digital Marketing Area (DMA)-level. Second, the platform-specific well-being differences among subpopulations in the primary dataset were probed and validated with similar inter-group differences at the individual level in the tertiary dataset. Thirdly, the main findings on the primary dataset were replicated with county-level depression diagnoses data for 2014–2018.

In all analyses, the linear mixed effects models were of the following form:

1 $$\begin{aligned} y_i,t = y_{i,t-1} + \alpha _i + \beta _1*Speed_i +\beta _2*SM_i,t,fb + \beta _3*SM_i,t,ig + \beta _4*SM_i,t,tw + \lambda _s + \varepsilon _its, \end{aligned}$$

where the key dependent variable, $y_i$ is the outcome, i.e., well-being, where a higher value indicates a higher appraisal of the quality of life. *i* indexes region (DMA or county), *s* indexes state, and *t* indexes year. $y_i$ was auto-regressed on its previous year’s value, $y_i,(t-1)$. $SM_i,t$ refer to the Facebook, Instagram, and Twitter visit frequencies in the same year by the region. $\lambda _s$, and $\alpha _i$ are state and region-level fixed effects.

Including location fixed effects can control for aggregate-level time shocks. Exogenous variations are accounted for by including $Speed_i,t$, a binary variable measuring whether an individual has access to broadband internet, signifying the quality of internet access. Before analysis, social media use and well-being were rescaled to [0,1] to allow for straightforward interpretation.

The lmerTest package in R^31^ (Version 3.1.3; https://cran.r-project.org/web/packages/lmerTest) was used for analysis with clustered standard errors as reported at the head of the columns in the Tables. In addition, respondent and regional sociodemographic correlates were also included in the models where “fixed effects” are indicated.

Before proceeding with any analysis, a sanity check on the data was conducted by comparing the well-being effects of self-reported general social media use in the primary dataset (at the county level) against a validation on the tertiary dataset (at the individual level) collected for an earlier period. The figures reporting the region-level and individual-level effects of general social media use in the supplementary materials, as well as the Fig. 2 reporting the effects of specific platform use, were generated using the ggplot2 package in R^63^ (Version 3.3.5; https://ggplot2.tidyverse.org/). Figure 3 and 4 were generated using the ggpubR package in R^29^ (Version 0.4; https://cran.r-project.org/web/packages/ggpubr).

## Supplementary Information

Supplementary Information.

## Data Availability

The figures and analyses in this paper are based on proprietary data and original data collected by the author.

## Code availability

Original code is available at the associated OSF repository for this project.
