# Supplementary material for: Cross-platform- and subgroup-differences in the well-being effects of Twitter, Instagram, and Facebook in the United States
Source: Sci Rep. 2022 Feb 28;12:3271. doi: 10.1038/s41598-022-07219-y (PMC8885821; doi:10.1038/s41598-022-07219-y)
Supplement: Supplementary file 1 — Supplementary Information. [file 41598_2022_7219_MOESM1_ESM.pdf]

Supplementary Materials to Cross-platform- and subgroup-differences in the well-being effects of Twitter, Instagram, and Facebook in the United States

Kokil Jaidka*

Department of Communications and New Media
National University of Singapore
Singapore

Question wording

Sharecare Gallup Well-being data

Well-Being Index According to the documentation provided with the Sharecare Gallup Well-being data, the Well-Being Index was calculated as an aggregate of 31 scored metrics from sub-indices (Purpose, Community, Physical, Financial and Social) and another 7 items related to subjective and affective well-being. It also included the following Life Satisfaction question: "Please imagine a ladder with steps numbered from zero at the bottom to ten at the top. The top of the ladder represents the best possible life for you and the bottom of the ladder represents the worst possible life for you. On which step of the ladder would you say you personally feel you stand at this time?"

Responses: 0 (Worst possible) - 10 (Best possible), Don't Know, Refused

The range of values of the final Well-Being Index was rescaled from 0 to 1, with a larger value reflecting higher well-being. The final measure reflected the average Well-Being Index of a county or Digital Marketing Area (DMA) per year.

% Depression diagnoses Have you ever been told by a physician or nurse that you have any of the following, or not? How about Depression?

- 1 Yes
- 2 No
- 3 (Don't know)
- 4 (Refused)

In the recoded variable for the analysis, responses 3 and 4 were discarded, The final measure reflected the fraction of respondents per county per year who reported that they had been diagnosed with depression.

County-level demographic information: Four demographic statistics (median age, % population over 65 years, % African American population and % Hispanic population, % While population), two socioeconomic status (SES) indicators (% population with a Bachelor's degree, logged average per capita income for individuals over the age of 25 in $) compiled by the US County Health Rankings and Roadmaps [1] were used as socio-demographic covariates.

*Corresponding author: Kokil Jaidka (jaidka@nus.edu.sg; ORCID linked to account on Manuscript Tracking System)

[1] www.countyhealthrankings.org

Simmons National Consumer Survey data

Individual social networking sites:

From 2015 onward, individual questions about the use of Facebook, Twitter, and Instagram were used to collect weekly visits and worded as follows: “Which of these websites have you visited in the last seven days?”

- Facebook.com
- Twitter.com
- Instagram.com

Monthly visit information was collected with the following question: “How many times did you visit each of these websites in the last 30 days?”

- 1-5
- 6-15
- 16 or more

A weighted average was used to calculate the average weekly and monthly visits of the entire population. These two variables were strongly correlated with each other. An arithmetic mean rescaled from 0 to 1 served as the final measure, where a higher value reflected more visits to an individual social media site.

Validation with Pew Research data

Pew Tracking Surveys are conducted twice a year on a stratified nationally representative sample. In each of eleven surveys between 2008 - 2013, respondents were asked about their satisfaction with life and their social media use. Additionally, information was collected about the quality of their internet access. Finally, respondents reported their age, gender, race, income, and education. Not all the surveys collected the state of residence, so this information could not be included in the analysis.

Question wording

Life Satisfaction

Overall, how would you rate the quality of life for you and your family today? Would you say it is... excellent, very good, good, fair or poor? Responses were reverse-coded before analysis:

- 1 Excellent
- 2 Very good
- 3 Good
- 4 Fair
- 5 Poor
- 8 (DO NOT READ) Don’t know
- 9 (DO NOT READ) Refused

Download speed

According to the documentation provided by Pew Research, the item wording for this question from December 2012 until May 2013 was - “At home, do you connect to the internet through a dial-up telephone line, or do you have some other type of connection, such as a DSL-enabled phone line, a cable TV modem, a wireless connection, or a fiber optic connection such as FIOS?”

December 2012 and earlier trend question wording included “T-1” as a read category. From September 2009 thru January 2010, the question asking about type of home internet connection (MODEM) was form split. MODEMA was asked of Form A respondents who use the internet from home. MODEMB was asked of Form B respondents who use the internet from home.

In the recoded variable used for analysis, the response reflects whether a respondent had (Download speed = 1) or did not have (Download speed = 0) at least a DSL connection at their home.

Social media use

According to the documentation provided by Pew Research, the item wording for this question from August 2011 until May 2013 was - “Do you ever use the internet to use a social networking site like Facebook, LinkedIn or Google Plus?"

From April 2009 through August 2011, the item wording for this question was “Do you ever use a social networking site like MySpace, Facebook or LinkedIn?" In December 2008, item wording was “Use a social networking site like MySpace or Facebook.” In August 2006, item wording was “Do you ever use an online social networking site like MySpace, Facebook or Friendster?” Prior to August 2006, item wording was “Do you ever use online social or professional networking sites like Friendster or LinkedIn?"

In the recoded variable used for analysis, the response reflects whether a respondent used (Social media use = 1) or did not use (Social media use = 0) social media.

Results

The well-being effects of general social media use

Table 1(a) reports the association between general social media use (visit frequency) and well-being at the county level for 20013-2018 in the primary dataset. The first column reports the results of a model that included only the dataset- and year-fixed effects. Next, column 2 included respondent fixed effects. According to Column 2, a 1% increase in social media use predicts an 0.6% rise in well-being (β = 0.64, $p < 0.01$).

The findings are validated against individual-level associations between social media use and life satisfaction between 2008-2013 in Table 1(b). The secondary data from Pew was not available after 2013 because Pew did not collect these survey items after 2013, and does not offer granular information about the social media platform being visited. The first column reports the results of a model that included only the dataset- and year-fixed effects. Next, column 2 included state fixed-effects. Finally, Column 3 included respondent fixed-effects.

Across models, the increase in social media use is associated with higher well-being. Furthermore, in all specifications, the relationship between the download speed and well-being was positive, although not significant after including respondent fixed effects, suggesting that physical barriers to social media access may also play a role when people self-select into using social media. According to Column 3, after controlling for sociodemographic and exogenous variations, a 1% increase in social media use predicts a 0.08% rise in well-being (β = 0.08, $p < 0.001$).

Figure 1(a) reports the association of general social media visits and well-being, as regressed against the county-level measures of satisfaction from 1.73 million responses to the Gallup-Sharecare Well-Being Index from 2014-2018 after including cross-lagged and fixed effects. The effects are pooled at the year-level. Figure 1(b) reports that the county-level findings bear out individual-level findings with Pew Track Surveys data from 2008-2013, where the increase in social media use is associated with higher well-being. By and large, the effects show the same positive trend over time.

Platform-specific well-being effects

The detailed results underlying Figure 2 are reported in Table 2. They are explained in the main text.

Platform-specific, group-level well-being effects

The detailed results underlying Figure 3 are reported in Table 3 which allows region-level inferences. Some of the many interesting differences and reported in the following paragraphs.

First, consider the effects of Facebook use on well-being across different demographic groups. The trends are steady, and across all groups the increase in Facebook use is typically associated with a statistically significant increase in well-being ($0.04 <= \beta <= 0.19$). Next, consider the group-level differences in the well-being effects of Twitter use. There is more variance here, but the statistically significant effects on well-being are negative and seen in counties with poor internet access (counties with broadband not available) (β = -0.08, $p < 0.05$), counties with a large population over 65 years (counties in the top quantile of a senior citizen population) (β = -0.17, $p < 0.01$), and counties with a large White population (β = -0.22, $p < 0.001$).

Finally, consider the group-level differences in the well-being effects of Instagram use. Counties with a large population under 18 years (counties in the top quantile of a population under the age of 18 years) report a 0.14% decrease in well-being with a 1% increase in Instagram use (β = -0.14, $p < 0.01$). On the

Figure 1. Estimates of the association between social media visit frequency and well-being with (a) the primary dataset at the county-level, and the (b) the secondary dataset at the individual-level. Estimates are pooled by year for (a) and by sample for (b). In all but one year and one sample, general social media visit frequency had a positive association with well-being (4.0<= β <=2.0 at the county-level; -0.05< β<0.2 at the individual-level)

(A) Primary dataset: Estimates of the association between county-level social media visit frequency and the Gallup-Sharecare Well Being Index, pooled by year. Data from the Simmons National Consumer Suvey, 2014-2018.

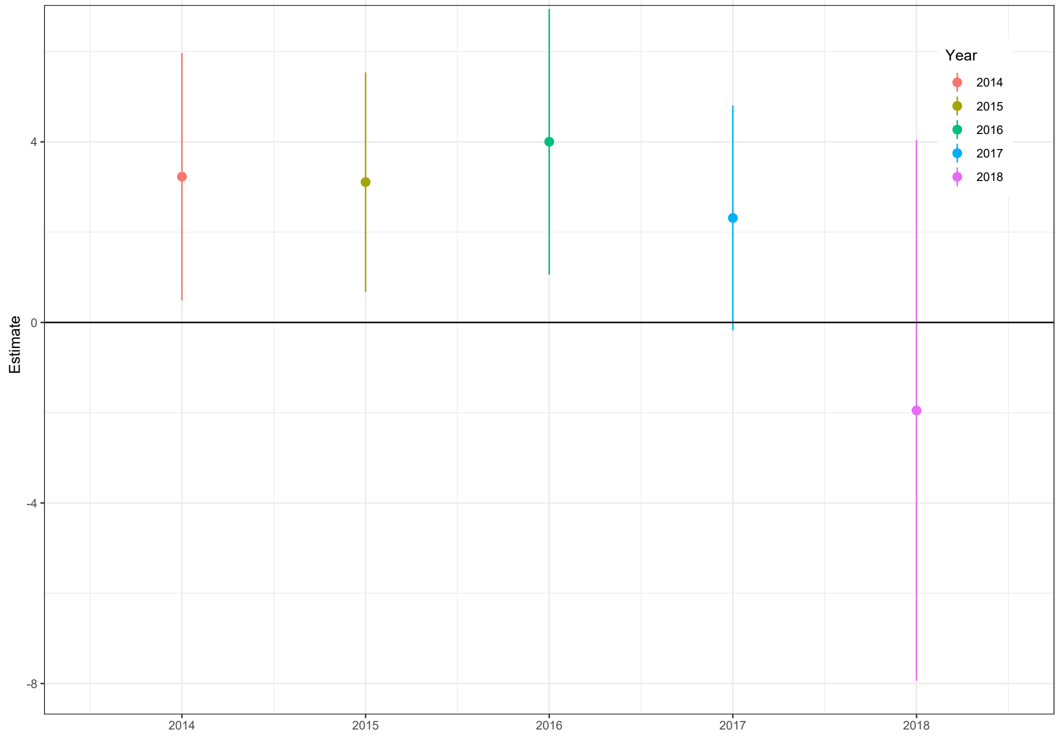

(B) Tertiary dataset: Estimates of the association between social media use and life satisfaction, pooled by dataset. Data from Pew Tracking Surveys, December 2008 - May 2013.

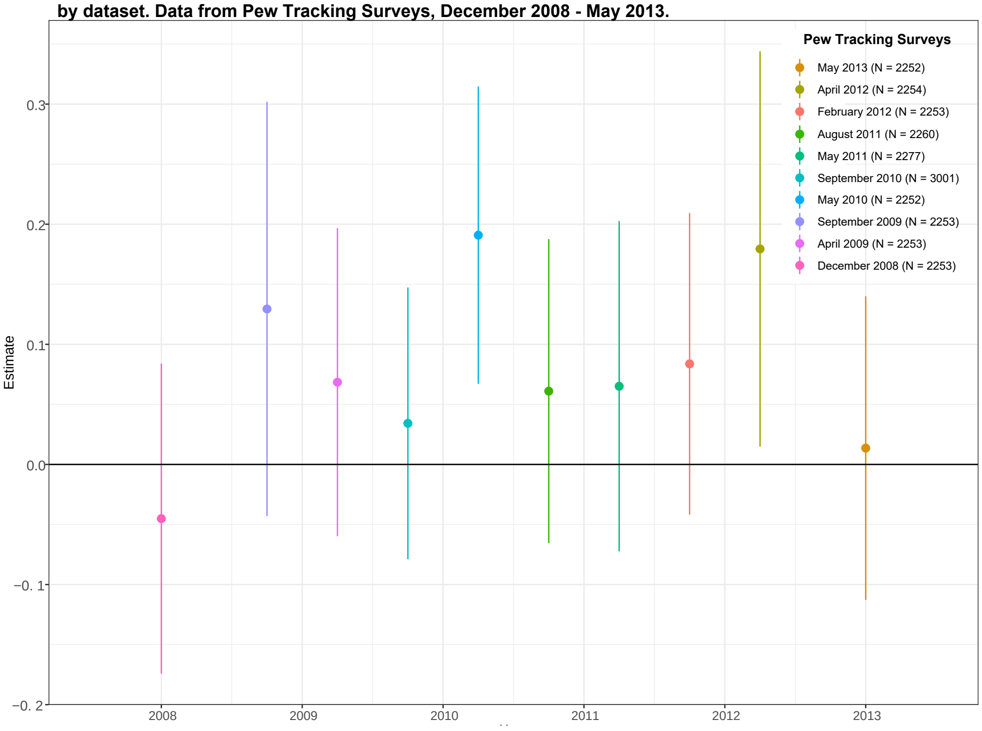

Table 1. The county-level effects of social media consumption on well-being (2014-2018, NCS data at county-level) are borne out in earlier indvidiual-level data from Pew (2008-2013, Pew data at the individual-level). All errors are clustered at the county-level. All models include socio-demographic factors related to age, sex, income, and race.

Primary dataset: National Consumer Survey
County-level data
vs. Gallup-Sharecare Well Being Index
β (Standard Error)
(2014 - 2018)

	**County fixed effects**	**Cross lagged & fixed effects**
**Download speed**	0.010**	0.013***
	(0.000)	(0.000)
**Social media visit frequency**	0.684***	0.637**
	(.168)	(0.190)
**Population under 18**		-0.002***
		0.000
**Population over 65**		0.003***
		0.000
**% Female population**		0.003
		0.000
**Household Income**	0.295***	0.249***
	0.019	0.019
**% White population**	0.000	0.000
	0.000	0.000
**% Black population**	-0.001	-0.001***
	0.000	0.000
**% Hispanic population**	0.000	0.001
	0.000	0.000
**Observations**	5161	4078
**Number of counties**	1222	1220
**Marginal R^2 m**	0.16	0.19
**Conditional R^2 c**	0.47	0.39

Secondary dataset: Pew Research
Individiual-level data
vs. Life Satisfaction
β (Standard Error)
(2008 - 2013)

	**Dataset + Year fixed effects**	**State fixed effects**	**Respondent fixed effects**
**Download speed**	0.25***	0.27***	0.04
	(0.02)	(0.02)	(0.03)
**Social media use**	0.11***	0.13***	0.08***
	(0.02)	(0.02)	(0.01)
**Age**	-	-	-.01***
	-	-	(0.00)
**Male**	-	-	-.07***
	-	-	(0.02)
**Income**	-	-	.12***
	-	-	(0.00)
**Education**	-	-	.06***
	-	-	(0.00)
**White**	-	-	-.03**
	-	-	(0.01)
**Hispanic**	-	-	.03
	-	-	(0.03)
**Black**	-	-	-.05+
	-	-	(0.03)
**Observations**	16,064	14,502	11,092
**Marginal R^2**	0.01	0.01	0.10
**Conditional R^2**	0.01	0.02	0.10

Note: *p<0.1; **p<0.05; ***p<0.01

Table 2. The county-level effects of platform-specific social media consumption on well-being (2014-2018, NCS data at county-level). All errors are clustered at the county-level. All models include socio-demographic factors related to age, sex, income, and race.

Primary dataset: National Consumer Survey
County-level data
vs. Gallup-Sharecare Well Being Index
β (Standard Error)
(2016 - 2018)

	**County fixed effects**	**Cross lagged & fixed effects**
**Download speed**	0.02***	.01***
	(0.00)	(0.00)
**Twitter visit frequency**	-0.06*	-0.05+
	(0.03)	(0.03)
**Instagram visit frequency**	-0.10***	-0.07*
	(0.03)	(0.03)
**Facebook visit frequency**	0.13***	0.11***
	(0.03)	(0.03)
**Observations**	3008	3008
**Number of counties**	1216	1216
**Marginal R^2 m**	0.18	0.26
**Conditional R^2 c**	0.46	0.26

Tertiary dataset: Google Trends
DMA-level data
vs. Gallup-Sharecare Well Being Index
β (Standard Error)
(2014 - 2018)

	**DMA fixed effects**	**Cross lagged & fixed effects**
**Download speed**	0.01	.01***
	(0.01)	(0.00)
**Twitter visit frequency**	0.07*	0.24***
	(0.03)	(0.06)
**Instagram visit frequency**	-0.16***	-0.12*
	(0.05)	(0.05)
**Facebook visit frequency**	0.21***	0.19***
	(0.04)	(0.04)
**Observations**	1020	816
**Number of DMAs**	204	204
**Marginal R^2 m**	0.27	0.34
**Conditional R^2 c**	0.64	0.56

Note: *p<0.1; **p<0.05; ***p<0.01

other hand, counties with a large White population (counties in the top quantile of the population of Whites, as per the National Census figures) report a 0.06% increase in well-being with a 1% increase in Instagram use (β = 0.06, p < 0.01). In contrast, counties in the top quantile of Black populations report a decrease (0.14%) in well-being for every 1% increase in Instagram use at the county level (β = -0.14, p < 0.01).

INSERT TABLE 1 HERE

Table 3. Primary dataset: Subgroup differences in the association of social media use with well-being (2016-2018, NCS data and Gallup-Sharecare at county-level). All models are cross-lagged and include all other covariates (age, gender, race, income, internet access quality, education) except the one that defines the subgroup.

Group	N | Counties	Effect size for predicting well-being		
		Estimate β (Standard Error)		
		Twitter visit frequency	Instagram visit frequency	Facebook visit frequency
All respondents	3008 | 1216	-0.07* (0.03)	-0.05+ (0.03)	0.11** (0.03)
Counties with good internet access	1226 | 559	-0.07+ (0.04)	-0.01 (0.04)	0.10* (0.04)
Counties with poor internet access	1782 | 876	-0.08+ (0.04)	-0.08* (0.04)	0.14* (0.04)
High income counties	1047 | 376	-0.06 (0.04	0.15 (0.03)	0.10* (0.04))
Low income counties	921 | 422	-0.07 (0.07)	-0.07 (0.07)	0.14* (0.07)
Counties with a large population under 18 years	989 | 400	-0.14** (0.05)	0.02 (0.04)	0.16** (0.04)
Counties with a large population over 65 years	964 | 433	-0.04 (0.07)	-.17** (0.06)	0.12+ (0.06)
Counties with a large White population	951 | 420	.06** (0.07)	-0.22*** (0.06)	0.19** (0.06)
Counties with a sizeable Black population (> 9.2%)	1031 | 396	-0.14** (0.05)	0.01 (0.04)	0.04 (0.04)
Counties with a sizeable Hispanic population (> 9.1%)	1024 | 390	-0.08* (0.04)	0.00 (0.03)	-0.00 (0.03)
Note:				+p<0.1; *p<0.05; **p<0.01 ; ***p<<0.001

A validation analysis was performed on the tertiary dataset. Group-level differences reported in Table 4 suggest that even at the individual level, greater social media use is predictive of higher well-being for high-income respondents (β = 0.09, p < 0.01), White respondents β = 0.12, p < 0.001, and those with good internet access (DSL connections in their homes) (β = 0.08, p < 0.001). On the other hand, higher social media use is predictive of lower well-being for Black respondents at the individual level (β = -0.13, p < 0.05).

Table 4. Secondary dataset: Subgroup differences in the association of social media use with well-being (2008-2013, Pew Research data at individual level). All models include all other covariates (age, gender, race, income, internet access quality, education) except the one that defines the subgroup.

Group	N	Effect size for predicting well-being	
		Estimate β	Standard Error
All respondents	11,092	0.08***	0.01
Males respondents	6,337	0.09**	0.03
Female respondents	5,930	0.07*	0.04
Low-income respondents	2,787	0.03	0.05
High-income respondents	3,879	0.09**	0.03
White respondents	8,793	0.12***	0.02
Black respondents	1,483	-0.13*	0.06
Hispanic respondents	1,151	0.06	0.07
Poor internet access	1,805	0.10+	0.06
Good internet access	9,287	0.08***	0.02
Note:		+p<0.1; *p<0.05; **p<0.01 ; ***p<<0.001	

Generalizability to mental health outcomes

Table 5 reports group-level differences in the association of social media use with self-reported depression diagnosis from the Gallup-Sharecare data, aggregated and averaged to the county-level. The associations at the high level lie in the opposite directions. However, drilling into demographic groups fulfills most expectations, where in all groups except counties with a larger Hispanic population, greater Facebook use is predictive of lower depression diagnoses (-0.22 <= β <= -0.10, p< 0.05). In all but the counties with broadband, greater Twitter is consistently predictive of higher depression diagnoses (0.11 <= β <= 0.37, p < 0.01). Instagram use predicts higher depression diagnoses in both, counties with good internet access and high income (0.11 <= β <= 0.12, p< 0.05) but also counties with no broadband access (β = 0.07, p < 0.05). Similar effects are seen for counties with either a large young or a large

senior citizen population ($0.14 <= \beta <= 0.21$, $p < 0.05$). There appear to be no differential effects of Instagram use in counties with more or fewer ethic populations.

Table 5. Primary dataset: Subgroup differences in the association of social media use with depression diagnoses (2016-2018, NCS data and Gallup-Sharecare data at county-level). All models are cross-lagged and include all other covariates (age, gender, race, income, internet access quality, education) except the one that defines the subgroup.

**Group**	**N | Counties**	**Effect size for predicting depression diagnoses<br>Estimate β (Standard Error)<br>Twitter visit frequency**	**Instagram visit frequency**	**Facebook visit frequency**
All respondents	3008 | 1216	-0.06^{*} (0.03)	-0.05^{+} (0.02)	0.12^{***} (0.02)
Counties with good internet access	1183 | 527	0.12^{***} (0.03)	0.10 (0.02)	-0.10^{*} (0.03)
Counties with poor internet access	1590 | 741	0.07^{*} (0.03)	0.15^{***} (0.03)	-0.09^{**} (0.03)
High income counties	1014 | 360	0.11^{*} (0.05)	0.11^{**} (0.04)	-0.16^{**} (0.05)
Low income counties	803 | 329	-0.01 (0.08)	0.44^{***} (0.08)	-0.22^{**} (0.08)
Counties with a large population under 18 years	912 | 347	0.14^{*} (0.06)	0.19^{***} (0.05)	-0.15^{*} (0.06)
Counties with a large population over 65 years	839 | 343	0.21^{*} (0.09)	0.24^{**} (0.08)	-0.09 (0.08)
Counties with a large White population	821 | 335	0.00 (0.09)	0.29^{***} (0.08)	-0.17^{*} (0.08)
Counties with a sizeable Black population (> 9.2%)	979 | 356	0.00 (0.06)	0.37^{***} (0.05)	0.00 (0.05)
Counties with a sizeable Hispanic population (> 9.1%)	980 | 355	0.03 (0.05)	0.27^{***} (0.04)	0.07^{+}(0.04)

Note: $^{+}p<0.1$; $^{*}p<0.05$; $^{**}p<0.01$; $^{***}p<<0.001$
